# Intrathecal baclofen therapy for severe spasticity in an adult with tethered cord syndrome: a case report

**DOI:** 10.1186/s13256-021-03049-0

**Published:** 2021-09-02

**Authors:** Yasutaka Takagi, Hiroshi Yamada, Hidehumi Ebara, Hiroyuki Hayashi, Satoshi Kidani, Kazu Toyooka, Yuji Ishino, Yoshiyuki Kitano, Aki Nakanami, Kenji Kagechika, Tetsutaro Yahata, Hiroyuki Tsuchiya

**Affiliations:** 1 Department of Orthopaedic Surgery, Tonami General Hospital, 1-61 Shintomi-cho, Toyama, 939-1395 Japan; 2 Department of Rehabilitation Medicine, Tonami General Hospital, 1-61 Shintomi-cho, Toyama, 939-1395 Japan; 3 Department of Rehabilitation Medicine, Toyama Prefectural Rehabilitation Hospital and Support Center for Children with Disabilities, 36 Shimoiino-machi, Toyama, 939-1395 Japan; 4 Department of Rehabilitation Medicine, Kanazawa University Hospital, 13-1 Takara-machi, Kanazawa, Ishikawa 920-8641 Japan; 5 Department of Orthopaedic Surgery, Graduate School of Medicine, Kanazawa University, 13-1 Takara-machi, Kanazawa, Ishikawa 920-8641 Japan

**Keywords:** Tethered cord syndrome, Spasticity, Baclofen, Intrathecal infusion

## Abstract

**Background:**

Patients with tethered cord syndrome often suffer severe spasticity. To the best of our knowledge, intrathecal baclofen (ITB) therapy in a patient with tethered cord syndrome has not been reported previously. We describe a case in which ITB therapy was useful for treating severe spasticity in an adult with tethered cord syndrome.

**Case presentation:**

We present the case of a 50-year-old Japanese woman with tethered cord syndrome and related conditions suffering from severe spasticity and pain in the lower limbs. She was born with a lumbosacral myelomeningocele, which was closed in the neonatal period. For 4–5 years before this presentation, spasticity in the lower limbs had been exacerbated without any obvious cause. She received rehabilitation and pharmacotherapy from a local doctor, but symptoms were unimproved, and her previous doctor referred her to this department. A test with 50 μg of intrathecally delivered baclofen showed total relief of spasticity and pain, so a pump was implanted for continuous baclofen delivery. During 24 months of follow-up, spasticity has remained under excellent control with baclofen at 38.5–41.0 μg/day.

**Conclusions:**

ITB therapy proved extremely effective in this adult with severe spasticity from tethered code syndrome.

## Background

Tethered cord syndrome (TCS) is a neurological abnormality in which the spinal cord is unable to slide normally inside the spinal canal. This disorder is mechanically caused by the effect of inelastic structures on the caudal spinal cord (filum terminale), limiting upward movement of the lumbosacral spinal cord [1]. TCS is characterized by symptoms and signs resulting from excessive tension on the spinal cord [2], including back and leg pain, change in bladder tone, change in motor or sensory levels, spasticity, deformities of the lower extremities, new onset or progression of scoliosis, and gait deterioration [2–7]. Symptomatic tethering of the spinal cord occurs in 2.8–27% of patients following primary myelomeningocele repair [3, 5, 6, 8, 9].

Intrathecal baclofen (ITB) therapy has been shown to substantially improve symptoms in most patients with severe spasticity due to traumatic spinal cord injury, multiple sclerosis, or cerebral paresis [10–16]. To the best of our knowledge, the effects of ITB therapy in adults with TCS have not been reported.

## Case presentation

**Fig. 1.**
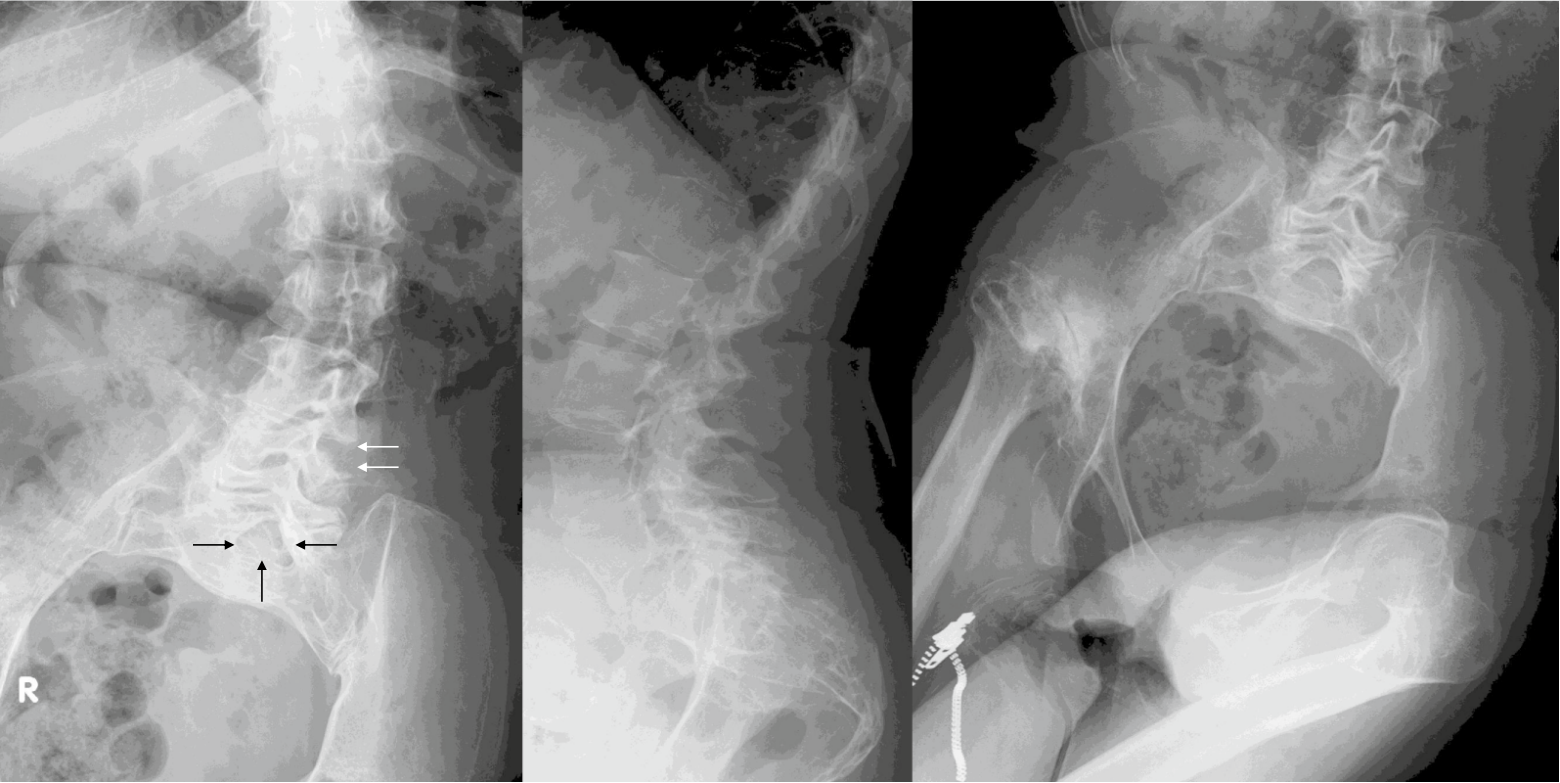
Preoperative X-ray image. Lumbar scoliosis, excessive lordosis, and osteoarthrosis of the right hip joint are evident. Left facet joint has disappeared (white arrow), and spina bifida occulta is apparent in the sacrum (black arrow)

**Fig. 2.**
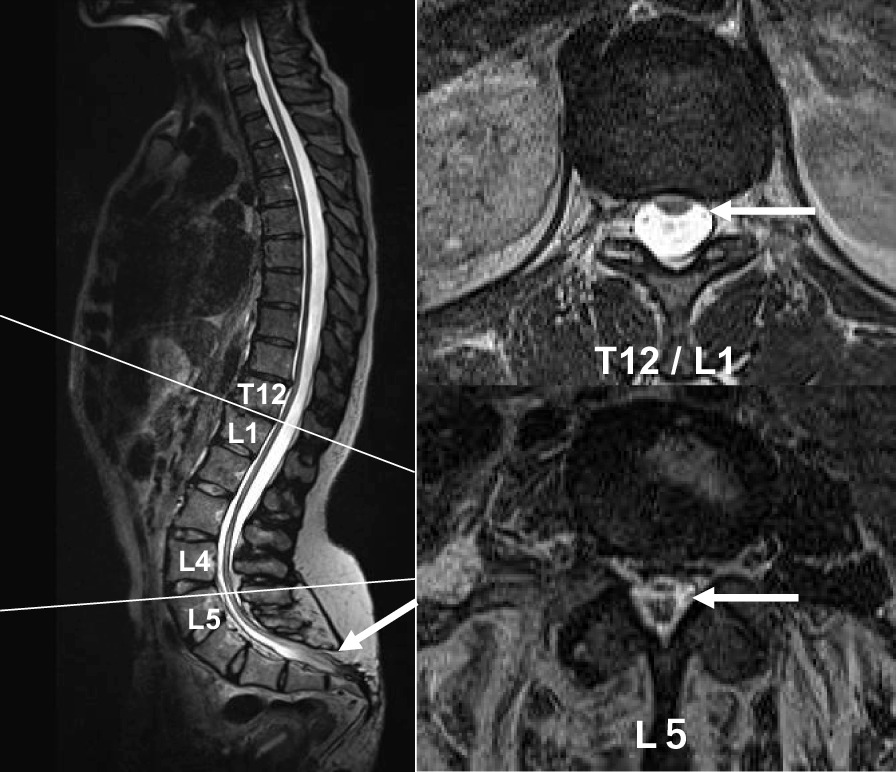
Magnetic resonance imaging with modified scoliosis. The spinal cord (white arrow) is located anteriorly in the thoracolumbar spinal canal and continues to the subcutaneous tissue of the sacrum

**Fig. 3:**
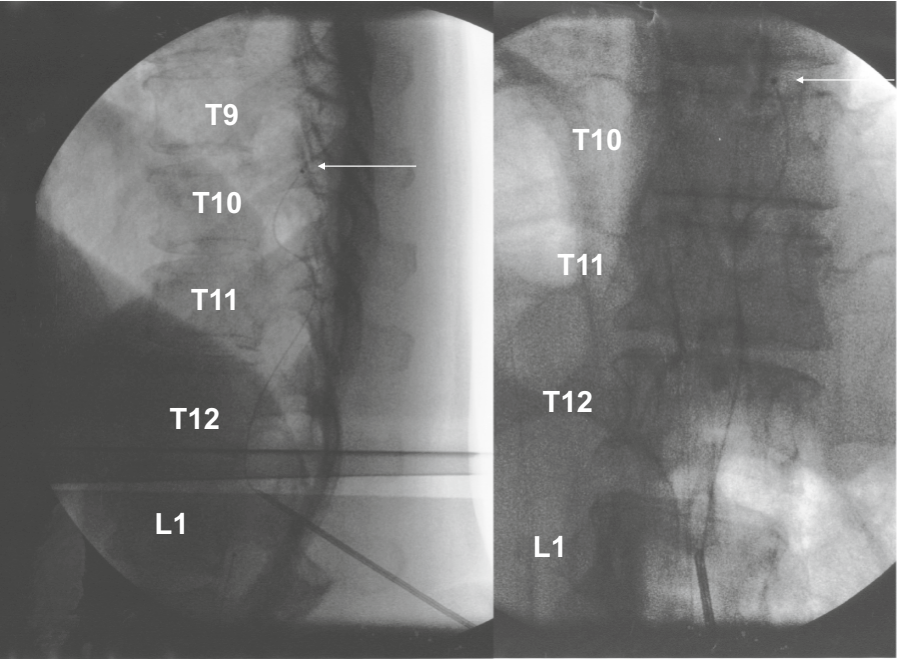
Intraoperative image. The catheter has entered the intrathecal space with the tip (white arrow) positioned over the T9–T10 interspace under fluoroscopy by paramedian puncture for catheter entry at the T12–L1 level

**Fig. 4:**
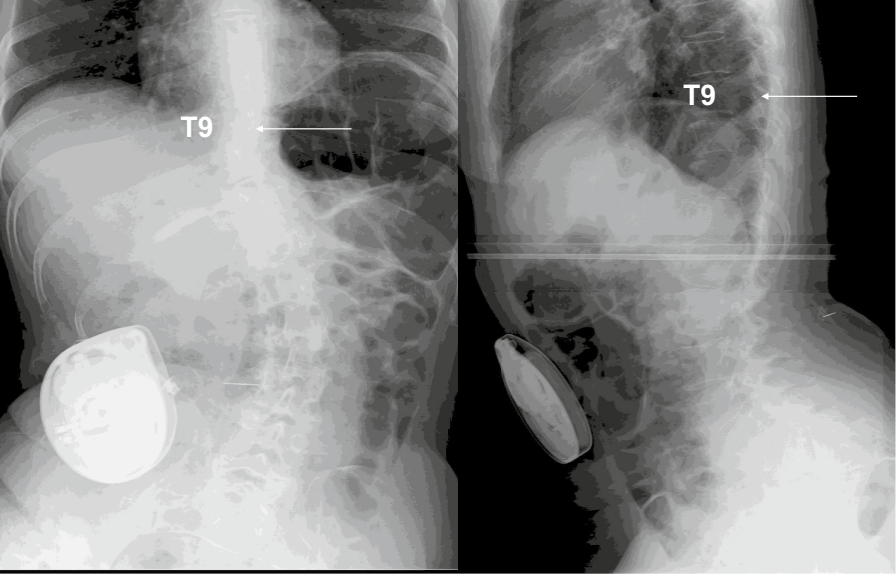
Postoperative X-ray. The intrathecal baclofen pump is seen in the right abdomen, and the tip (white arrow) of the catheter is placed at the T12–L1 level

We present the case of a 50-year-old Japanese woman with TCS and related conditions suffering from severe spasticity and pain in the lower limbs. She was born in February 1969 with a lumbosacral myelomeningocele. Surgery to close the myelomeningocele was successfully performed in the neonatal period. For 4–5 years before the current presentation, the patient had experienced exacerbating spasticity in the lower limbs without any evident cause. She had received rehabilitation and pharmacotherapy from a local doctor, but symptoms remained unimproved, and she was referred to her previous doctor, who in turn introduced her to this department. On presentation, spasticity of the lower limbs was very severe. The degree of spasticity was evaluated using the Modified Ashworth Scale (MAS) (Table 1) [17]. MAS was evaluated for eight sites: the hip extensors (4/4), hip adductors (4/4), knee extensors (3/3), and ankle plantar flexors (3/3) in both lower limbs. She was unable to sleep in bed in a supine position due to severe spasticity of both legs. Touching her thighs resulted in painful muscle spasms. She was able to walk with two crutches. X-ray images showed lumbar scoliosis, excessive lordosis, and osteoarthrosis of the right hip joint. The X-ray image showed that the left L3/4 facet joint had disappeared and that spina bifida occulta was evident in the sacrum (Fig. 1). Magnetic resonance imaging (MRI) with modified scoliosis revealed the spinal cord located anteriorly in the thoracolumbar spinal canal and continuing to the subcutaneous tissue of the sacrum (Fig. 2). Spasticity gradually increased in severity over 4–5 years, and the decision was made to evaluate the efficacy of ITB. Baclofen was administered intrathecally as a single dose of 50 μg, yielding an excellent response with complete resolution of spasticity. She consented to implantation of a programmable pump that could deliver ITB continuously. The catheter was introduced into the intrathecal space with the tip positioned over the T9–T10 interspace under fluoroscopy by paramedian puncture for catheter entry at the T12–L1 level (Fig. 3). A pump for continuous delivery of baclofen (SynchroMed II, Medtronic, Inc., Minneapolis, MN, USA) was implanted in the right abdomen (Fig. 4). After ITB pump implantation, spasticity was greatly improved, and MAS at the eight sites was as follows: hip extensors, 1/1; hip adductors, 1/1; knee extensors, 1/1; and ankle plantar flexors, 1/1. During 24 months of follow-up, spasticity has remained under excellent control on baclofen at 38.5–41.0 μg/day without adverse effects. The patient was able to sleep in bed in the supine position and was able to walk with two crutches without feeling weak after ITB pump implantation. ITB therapy proved extremely effective for improving spasticity in this adult with severe spasticity from TCS.

**Table 1:** Modified Ashworth Scale for grading spasticity

Grade	Description
0	No increase in muscle tone
1	Slight increase in muscle tone, manifested by a catch and release or by minimal resistance at the end of the range of motion when the affected part(s) is moved in flexion or extension
1+	Slight increase in muscle tone, manifested by a catch, followed by minimal resistance throughout the remainder (less than half) of the range of motion
2	More marked increase in muscle tone through most of the range of motion, but affected part(s) easily moved
3	Considerable increase in muscle tone, passive movement difficult
4	Affected part(s) rigid in flexion or extension

## Discussion

Since the first report in 1984 that ITB therapy could eliminate spasticity of a spinal cord origin, numerous studies in the United States and Europe have corroborated those results [10–16]. Investigators have documented not only marked decreases in abnormal tone and spasms with baclofen use, but also associated improvements in activities of daily living, sleep patterns, and bladder function [10–16].

Bergenheim *et al.* reported ITB therapy for spasticity in a child with myelomeningocele [18]. To the best of our knowledge, ITB therapy has not been reported in an adult with TCS. This appears to be the first description of ITB therapy for severe spasticity in an adult with TCS.

TCS is characterized by symptoms and signs resulting from excessive tension on the spinal cord [2], including back and leg pain, change in bladder tone, changes in motor or sensory levels, spasticity, deformities of the lower extremities, new onset or progression of scoliosis, and gait deterioration [2–7]. Symptomatic tethering of the spinal cord following primary myelomeningocele repair occurs in 2.8–27% of patients [3, 5, 6, 8, 9].

The surgical outcomes of untethering remain controversial. Untethering surgery has varying effects on different symptoms, making it difficult to restore bladder function while relieving pain. Given the possible high risk of recurrence, further exploration of the indications and timing of untethering surgery is necessary [19].

Baclofen is a gamma-aminobutyric acid (GABA) receptor agonist and is the most frequently used agent for the treatment of spasticity. However, its use has been limited due to systemic side effects such as drowsiness, confusion, and headache [20]. ITB therapy can directly and effectively control spasticity by selectively acting as a GABA receptor agonist in the compartment of the spinal cord, with fewer systemic side effects [10–13, 16]. Moreover, while orthopedic musculoskeletal surgery and selective posterior rhizotomy are irreversible surgeries, ITB therapy is reversible and allows continuous control of spasticity [21]. ITB therapy has been reported to significantly reduce spasticity that has proven refractory to oral medications and botulinum toxin treatment [10–13, 16].

The risk of spinal cord injury should be noted in puncture and catheter placement for TCS with spinal deformity. Due to excessive lordosis and degenerative scoliosis of the lumbar spine, puncture and catheter placement in the lower lumbar spine are very risky. Since the spinal cord was located anteriorly in the thoracolumbar spinal canal in this case, careful attention was paid to spinal cord injury, and puncture and catheter placement were performed at the level of the thoracolumbar junction. If puncture and catheter placement prove difficult, a change to open laminectomy may be necessary for puncture and catheter placement.

ITB therapy was performed for lower limb spasticity due to TCS in this case, resulting in marked improvements. To the best of our knowledge, this is the first report of ITB therapy for severe spasticity in an adult with TCS.

## Conclusions

ITB therapy was very effective in improving spasticity in an adult with severe spasticity from TCS. This is the first report of ITB therapy for severe spasticity in an adult with TCS.

## Data Availability

Medical imaging data will not be shared because it is not fully anonymous.

## Abbreviations

ITB: Intrathecal baclofen
TCS: Tethered cord syndrome
MRI: Magnetic resonance imaging
MAS: Modified Ashworth Scale
GABA: Gamma-aminobutyric acid
